# Reproducibility and Sensitivity of High-Throughput Sequencing (HTS)-Based Detection of Citrus Tristeza Virus and Three Citrus Viroids

**DOI:** 10.3390/plants11151939

**Published:** 2022-07-26

**Authors:** Rachelle Bester, Chanel Steyn, Johannes H. J. Breytenbach, Rochelle de Bruyn, Glynnis Cook, Hans J. Maree

**Affiliations:** 1 Citrus Research International, P.O. Box 2201, Matieland 7602, South Africa; rachelle@sun.ac.za; 2 Department of Genetics, Stellenbosch University, Private Bag X1, Matieland 7602, South Africa; rochelle@cri.co.za; 3 Citrus Research International, P.O. Box 28, Nelspruit 1200, South Africa; chanel@cri.co.za (C.S.); kobus@cri.co.za (J.H.J.B.); glynnis@cri.co.za (G.C.); 4 Department of Plant Pathology, Stellenbosch University, Private Bag X1, Matieland 7602, South Africa

**Keywords:** CTV, hop stunt viroid (HSVd), citrus dwarfing viroid (CDVd), citrus exocortis viroid (CEVd), next-generation sequencing, repeatability, specificity

## Abstract

The credibility of a pathogen detection assay is measured using specific parameters including repeatability, specificity, sensitivity, and reproducibility. The use of high-throughput sequencing (HTS) as a routine detection assay for viruses and viroids in citrus was previously evaluated and, in this study, the reproducibility and sensitivity of the HTS assay were assessed. To evaluate the reproducibility of HTS, the same plants assayed in a previous study were sampled again, one year later, and assessed in triplicate using the same analyses to construct the virome profile. The sensitivity of the HTS assay was compared to routinely used RT-PCR assays in a time course experiment, to compensate for natural pathogen accumulation in plants over time. The HTS pipeline applied in this study produced reproducible and comparable results to standard RT-PCR assays for the detection of CTV and three viroid species in citrus. Even though the limit of detection of HTS can be influenced by pathogen concentration, sample processing method and sequencing depth, detection with HTS was found to be either equivalent or more sensitive than RT-PCR in this study.

## 1. Introduction

The first step in disease management is the accurate detection of the etiological agent. Only after a detection assay has been developed for a specific pathogen can incidence and prevalence be investigated, and a control strategy can be devised. The sensitivity of the detection assay is a key determinant of how effective that assay will be to arrest the spread of the pathogen. Viruses and viroids can threaten the sustainability of agricultural crop production, and the prevention of their introduction and spread are best managed by the production of healthy and pathogen-free plant propagation material. Quarantine and certification programs established to prevent the spread of plant diseases rely on either biological indexing, serological-based assays, or nucleic-acid-based assays. These assays can be sequence dependent or targeted towards a specific pathogen limiting the detection of diverse variants or unknown pathogens. The many advances of high-throughput sequencing (HTS) for the detection of plant pathogens have been highlighted previously [1,2,3,4,5]. HTS is a technology broadly used in plant virus detection due to the panoptic approach and potential shorter turnaround time by simultaneous screening for multiple pathogens. Therefore, many countries are investigating how to best apply HTS in certification programs [4,6,7]. If HTS can meet all the validation criteria (sensitivity, specificity, reproducibility, and repeatability) [3], it will be possible to identify plant material devoid of detectable pathogens, and allow the early propagation of this material without accruing additional risk. This plant material could then be propagated with provisional status in designated areas pending the completion of specified validations. It is important to evaluate novel methods such as HTS through direct comparisons to existing, validated molecular assays, for its comparable ability to detect pathogens of interest. Each step in a detection assay, from sampling to result visualization, can influence the final diagnostic call [7]. It is therefore essential to determine the limits of detection for each assay. Attempts to compare the sensitivity of HTS to other molecular assays were made and it was shown that HTS can have a similar level of sensitivity [8,9,10,11].

In this study, the reproducibility and sensitivity of an HTS detection assay was directly compared to the RT-PCR assays of the standard operating procedures (SOP) of the Citrus Improvement Scheme (CIS) of southern Africa for the detection of citrus viruses and viroids. This study builds on the previous research by Bester et al. [7] where HTS assay variation in the detection of citrus viruses and viroids was measured. The Illumina HTS pipeline used in Bester et al. [7] was applied to the same plant material assayed with RT-PCR in a time course experiment over seven months to evaluate the sensitivity of each method as natural virus and viroid accumulation in the plant progressed.

## 2. Results

### 2.1. Reproducibility

The reproducibility of the HTS assay was assessed by re-sampling the four biological replicate plants (*C. sinensis* cv. ‘Madam Vinous’, sweet orange graft inoculated with a suite of viruses) from Bester et al. [7] in triplicate, one year later and applying the CTAB/Illumina pipeline. On average 25 million paired-end reads were received per sample from the Illumina HTS and after stringent quality trimming more than 96% of the data per sample was retained (Supplementary File S1) which is comparable to what was obtained in 2020 [7] (Supplementary File S1). The data was de novo assembled using SPAdes into an average of 74,171 scaffolds with an average N50 of 1831 nt (Supplementary File S1). Based on the BLASTn analysis of the scaffolds, the complete viral and viroid profiles expected for all the biological and technical replicates were obtained. No scaffolds of virus or viroid origin were identified in the three healthy plant technical replicates.

**Figure 1:**
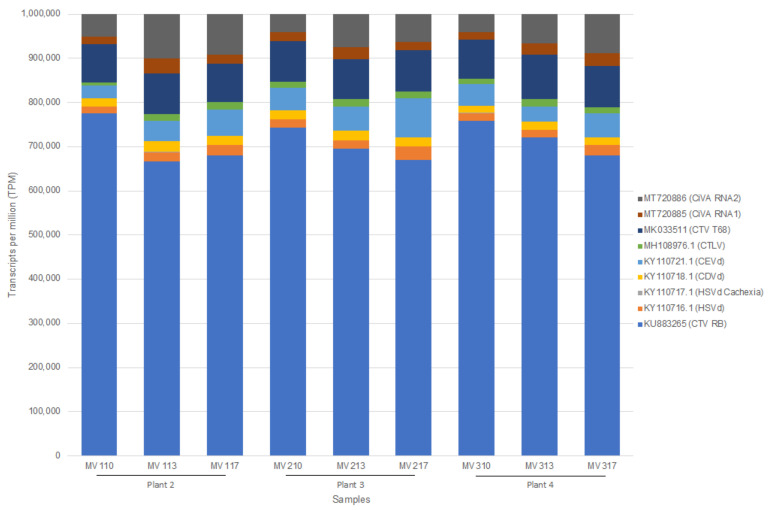
Stacked column chart displaying the transcripts per million (TPM) ratio for each pathogen reference analyzed. The ratios of the TPM count of each pathogen relative to the TPM count of the other pathogens are displayed for each sample subjected to the CTAB/Illumina HTS protocol (CTV: citrus tristeza virus; CiVA: citrus virus A; CTLV: citrus tatter leaf virus; HSVd: hop stunt viroid; CDVd: citrus dwarfing viroid; CEVd: citrus exocortis viroid). MV110, MV113, and MV117 represent RNA extraction technical replicates from infected plant 2 (*C. sinensis* cv. ‘Madam Vinous’, sweet orange), MV210, MV213 and MV217 represents RNA extraction technical replicates from infected plant 3 (*C. sinensis* cv. ‘Madam Vinous’, sweet orange) and MV310, MV313, and MV317 represent RNA extraction technical replicates from infected plant 4 (*C. sinensis* cv. ‘Madam Vinous’, sweet orange).

As with the 2020 data, reads were mapped to reference genomes of the expected viruses and viroids including citrus tristeza virus (CTV) genotype RB, citrus virus A (CiVA) RNA 1 and 2, citrus tatter leaf virus (CTLV), hop stunt viroid (HSVd), citrus dwarfing viroid (CDVd) and citrus exocortis viroid (CEVd). Concurrently, reads were also mapped to the non-target CTV genotype T68 and the Cachexia causing variant of HSVd. The expected virome was detected using read mapping with more than 99% genome coverage (percentage of bases covered on the reference genome) for CTV genotype RB (KU883265), CTLV (MH108976.1), HSVd (KY110716.1), CEVd (KY110721.1), and CiVA RNA1 (MT720885) and CiVA RNA2 (MT720886). CDVd (KY110718.1) had a genome coverage ranging from 95 to 100% between samples. Read mapping to the non-target CTV genotype T68 showed an average genome coverage of only 61% and to the non-target Cachexia causing variant of HSVd only 32% genome coverage was obtained on average. The transcripts per million (TPM) values were used to compare the proportion of reads that mapped to a virus and viroid accession in each sample. As shown in 2020, CTV had the highest TPM count with similar proportions of reads mapping to each of the other pathogens (Figure 1). A significant association between the two sampling time points was observed based on the TPM values of the pathogen sequences per sample as measured by the Spearman’s rank correlation test. Correlation coefficients (Rho) of equal or above 0.75 and *p*-values of <0.05 were calculated (Supplementary File S2). No significant read mapping to reference genomes was obtained from the three technical replicates of the healthy plant (only MVH3 had 16 CTV reads).

**Figure 2:**
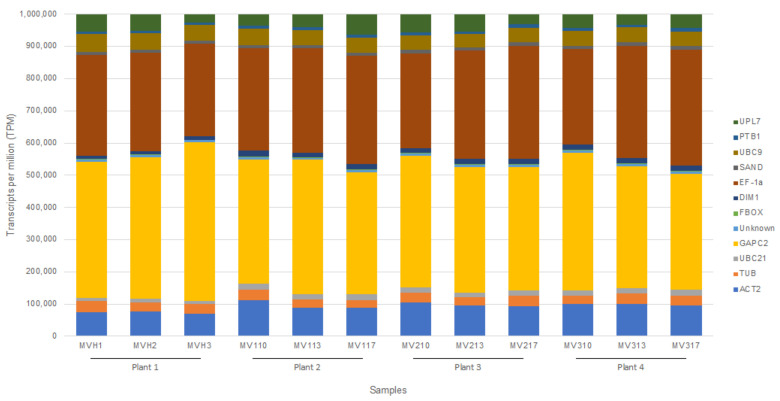
Stacked column chart displaying the transcripts per million (TPM) values for each reference gene analyzed. The ratios of the TPM count of each gene relative to the TPM count of the other genes are displayed for each sample subjected to the CTAB/Illumina HTS protocol (SAND: SAND family protein; DIM1: DIM1 homolog/YLS8; Unknown protein: Citrus sinensis carnosine N-methyltransferase-like transcript; FBOX: F-box family protein; UPL7: Ubiquitin-protein ligase 7; UBC21: Ubiquitin-conjugating enzyme 21; PTB1: Polypyrimidine tract-binding protein 1; GAPC2: Glyceraldehyde-3-phosphate dehydrogenase C2; UBC9: Ubiquitin conjugating enzyme 9; ACT2: Actin-2; EF-1a: Elongation factor 1-alpha; TUB: beta-Tubulin). MVH1, MVH2, and MVH3 represent RNA extraction technical replicates from healthy plant 1 (*C. sinensis* cv. ‘Madam Vinous’, sweet orange), MV110, MV113, and MV117 represent RNA extraction technical replicates from infected plant 2 (*C. sinensis* cv. ‘Madam Vinous’, sweet orange), MV210, MV213, and MV217 represent RNA extraction technical replicates from infected plant 3 (*C. sinensis* cv. ‘Madam Vinous’, sweet orange), and MV310, MV313, and MV317 represent RNA extraction technical replicates from infected plant 4 (*C. sinensis* cv. ‘Madam Vinous’, sweet orange).

Reads were also mapped to the 12 *C. sinensis* reference genes to identify biological and technical variation as in Bester et al. [7]. The same genes that had the highest TPM count across all samples in 2020, again had the highest TPM count (GAPC2, EF-1a and ACT2 (Figure 2)) and the genes with the lowest TPM count across all samples were again FBOX and the unknown gene, predicted as a *C. sinensis* carnosine N-methyltransferase-like transcript (Figure 2). No significant variation in TPM counts between the healthy and infected samples per gene were observed. However, based on the Kruskal-Wallis Rank Sum Test, the TPM count ratio of GAPC2 to ACT2 was significantly higher compared to the TPM count ratio in 2020 (*p*-value > 0.05). The TPM count ratio of EF-1a to UPL7 was also lower in 2020 (*p*-value > 0.05), but the UPL7 to UBC9 ratio was higher in 2020 (*p*-value > 0.05). A significant association between the two sampling time points was observed based on the TPM values of the gene sequences per sample as measured by the Spearman’s rank correlation test. Correlation coefficients (Rho) of equal or above 0.85 and *p*-values of <0.05 were calculated (Supplementary File S2).

### 2.2. Sensitivity

The sensitivity of the Illumina HTS assay was assessed by comparing RT-PCR results obtained at four time points (RT-PCR sample set) from six ‘Madam Vinous’ and six ‘Mexican Lime’ patch-graft inoculated with CTV, CiVA, HSVd, CDVd, CEVd, and ‘*Candidatus* Liberibacter africanus’ (CLaf) with the de novo assembly and read mapping results obtained from RNA from the HTS sample set from the same plants.

**Figure 3:**
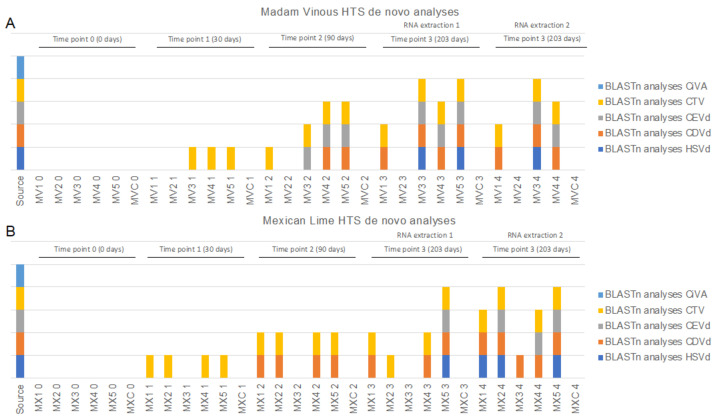
Stacked column chart displaying the virome profile of Madam Vinous (**A**) and Mexican Lime (**B**) plants at different time points constructed with BLASTn results of de novo assembled contigs of HTS reads. Each column represents a plant (MV1–5 or MX1–5) from which RNA was extracted 5 times (0–4). Extractions 3 and 4 were extracted both on 203 days post inoculation, but with different RNA extraction methods (RNA extraction 1: CTAB with LiCl precipitation; RNA extraction 2: CTAB extraction with ethanol precipitation). Samples MVC and MXC represent the healthy controls. Sample MV5 (extraction 4) was not evaluated with HTS. (CTV: citrus tristeza virus, Ci-VA: citrus virus A; HSVd: hop stunt viroid; CDVd: citrus dwarfing viroid; CEVd: citrus exocortis viroid).

**Figure 4:**
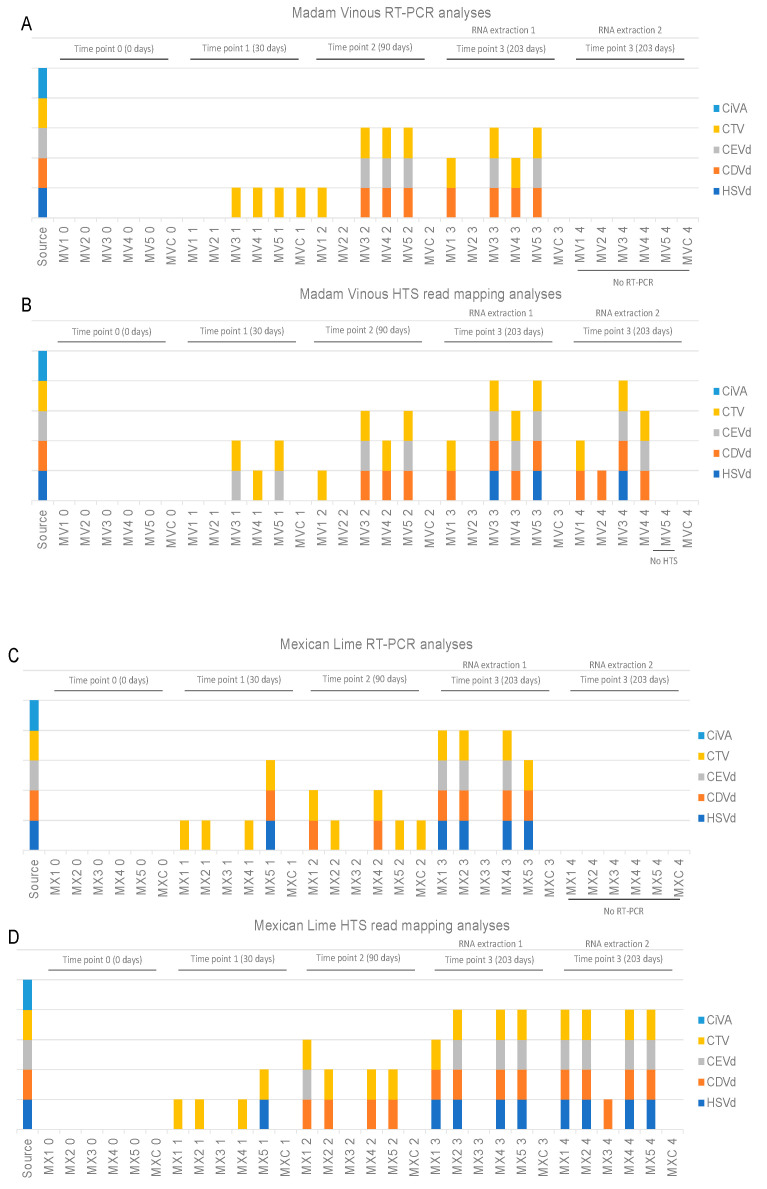
Stacked column chart displaying the virome profile of Madam Vinous (**A**,**B**) and Mexican Lime (**C**,**D**) plants at different time points constructed with RT-PCR (**A**,**C**) and HTS read mapping analyses (**B**,**D**). Each column represents a plant (MV1–5 or MX1–5) from which RNA was extracted 5 times (0–4). Extractions 3 and 4 were extracted both on 203 days post inoculation, but with different RNA extraction methods (RNA extraction 1: CTAB with LiCl precipitation; RNA extraction 2: CTAB extraction with ethanol precipitation). Samples MVC and MXC represent the healthy controls. Sample MV5 (extraction 4) was not evaluated with HTS. Samples MV1–5 (extraction 4) and samples MX1–5 (extraction 4) were not evaluated with RT-PCR (CTV: citrus tristeza virus; CiVA: citrus virus A; HSVd: hop stunt viroid; CDVd: citrus dwarfing viroid; CEVd: citrus exocortis viroid).

On average 23 million paired-end reads were received per sample from the Illumina HTS and after stringent quality trimming 96% of the data per sample was retained (Supplementary File S3). The data were de novo assembled using SPAdes into an average of 60,284 scaffolds with an average N50 of 1812 nt (Supplementary File S3). Based on the BLASTn analysis of the scaffolds, read mapping and RT-PCRs, no virus or viroid sequences were identified in the uninoculated samples at each time point or in any of the inoculated samples at time point 0 (Supplementary File S3, Figure 3). At time point 1 (30 days post inoculation) only CTV was detected with de novo assembly/BLASTn analyses (Figure 3). The RT-PCR assays produced the same results for the Madam Vinous’ plants at time point 1. With read mapping, CEVd was additionally detected in two ‘Madam Vinous’ samples at time point 1 (Figure 4B). In both the ‘Mexican Lime’ and ‘Madam Vinous’ plants, there was one sample (MV2 and MX3) that remained negative for all tested pathogens with both the de novo assembly/BLAST and read mapping pipelines and with RT-PCR, except when the RNA was extracted using the second RNA extraction protocol with acidic phenol phase separations and precipitation with ethanol at 203 days post inoculation (Figure 4). In all samples, the same number or more pathogens were detected with HTS compared to RT-PCR, except for four samples where the inverse was observed (Figure 4). In the healthy control ‘Madam Vinous’ sample of the 30 days post inoculation time point (MVC 1) and the healthy control ‘Mexican Lime’ sample of the 90 days post inoculation time point (MVC 2) CTV was detected via RT-PCR with a faint amplicon (Figure 4A); however, RNA extraction or PCR contamination is the most probable explanation, since these seedlings were grown from seed in an insect-free greenhouse and tested negative in subsequent assays. Two ‘Mexican Lime’ plants, MX5 (time point 1) and MX1 (time point 3, RNA extraction 1), additionally tested positive with only RT-PCR for CDVd and CEVd, respectively (Figure 4C and Figure 4D). No plants tested positive for CLaf or CiVA at time point 4 (203 days post inoculation). The HTS library construction for MV5 (time point 3, RNA extraction 2) was not successful and no sequencing data was obtained for this sample.

**Figure 5:**
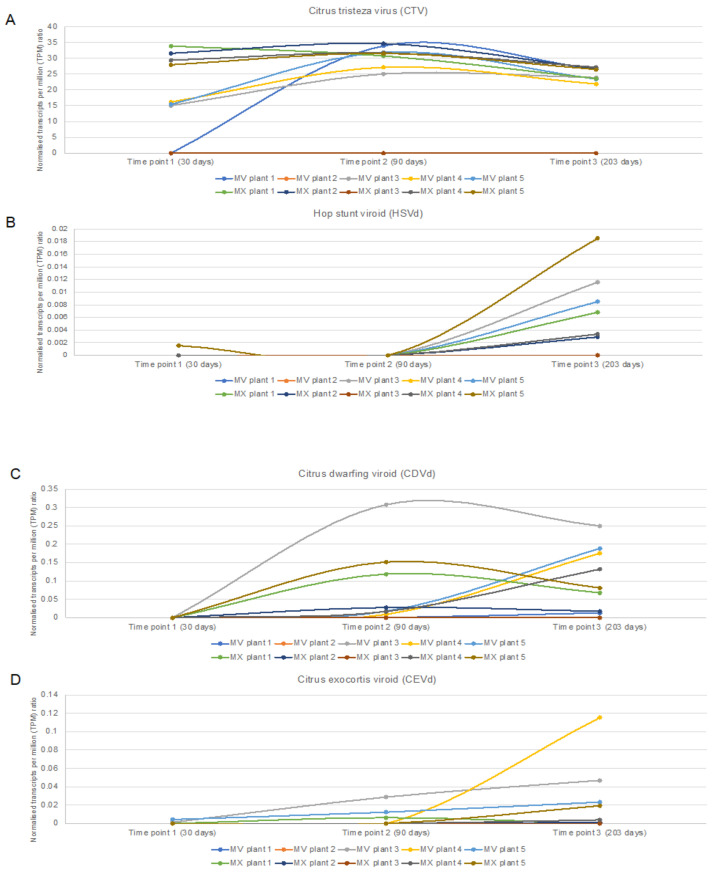
Line diagram displaying the relative pathogen concentration (normalized transcripts per million (TPM) ratio) from time point 0 (0 days post inoculation) to time point 3 (203 days post inoculation) for citrus tristeza virus (CTV) (**A**), Hop stunt viroid (HSVd) (**B**), citrus dwarfing viroid (CDVd) (**C**), and citrus exocortis viroid (CEVd) (**D**). Five plants per cultivar were evaluated (MV: ‘Madam Vinous’; MX: ‘Mexican Lime’).

**Figure 6:**
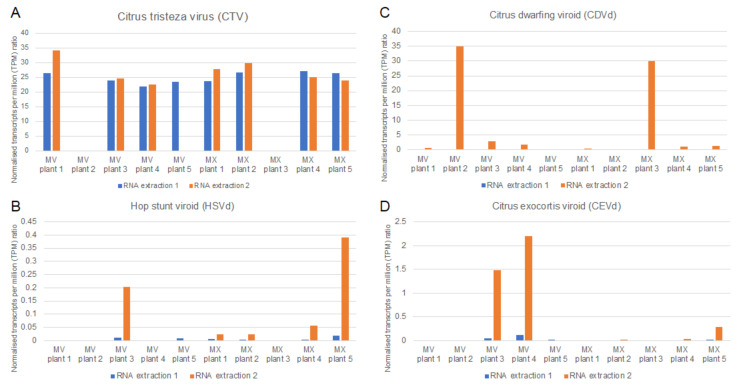
Clustered column chart displaying the relative pathogen concentration (normalized transcripts per million (TPM) ratio) at time point 3 (203 days post inoculation) for citrus tristeza virus (CTV) (**A**), Hop stunt viroid (HSVd) (**B**), citrus dwarfing viroid (CDVd) (**C**) and citrus exocortis viroid (CEVd) (**D**) obtained from RNA extracted with two different RNA extraction methods (RNA extraction 1: CTAB with LiCl precipitation; RNA extraction 2: CTAB extraction with ethanol precipitation) from five plants per cultivar (MX: ‘Mexican Lime’; MV: ‘Madam Vinous’).

The geometric mean of the reference genes’ TPM values, obtained by mapping the HTS data to the 12 *C. sinensis* reference genes, was used to normalize the TPM value of each pathogen to calculate a relative pathogen-specific concentration to compare between samples. For both the ‘Mexican Lime’ and ‘Madam Vinous’ plants, there was a gradual increase over time in pathogens detected at each time point as the different pathogen infections established in the plant over time (Figure 5). The direct comparison between two different RNA extractions from the same plant material at 203 days post inoculation (time point 3) showed that RNA extraction can have an impact on the detection of viroids with HTS (Supplementary File S3, Figure 6). The same viroid profile was detected in both the RNA extracts from the ‘Madam Vinous’ plants using a de novo/BLASTn analyses; however, more viroids could be detected in the RNA extracts from the ‘Mexican Lime’ plants obtained with RNA extraction method 2 using the same analyses (Supplementary File S3). With RNA extraction method 2, more viroids were also detected in the Mexican Lime plants using a read mapping pipeline. Analyses of the relative concentration of the different pathogens at time point 3 also showed that the viroid concentration as determined with normalized TPM values, was higher in the RNA extract obtained with RNA extraction 2 compared to RNA extraction 1 (Figure 6). For CTV, the RNA extraction method did not have a significant impact on the normalized concentration of the virus in the samples analyzed (Figure 6). Despite the overall increase in viroid detection from time point 1 to 3, the viroid detection was erratic, with specific viroids detected at earlier time points and not in subsequent time points in a specific sample (Figure 4A–D and Figure 5).

## 3. Discussion

Two parameters critical to the credibility of a pathogen detection assay were evaluated in this study. The use of HTS as a routine detection assay for viruses and viroids in citrus was previously evaluated [7]. To evaluate the reproducibility of the results obtained with HTS, the same plants evaluated in the previous study [7] were sampled again, one year later, and analyzed in triplicate using the same analyses to construct their virome profile. The second parameter investigated in the present study was sensitivity compared to routinely used RT-PCR assays. The sensitivity measurement was conducted in a time course experiment to also compensate for natural pathogen accumulation in plants over time.

The HTS pipeline applied in this study produced similar or more consistent results, one year later, for the same plants. The full virome profile was constructed and both the de novo assembly/BLASTn and read mapping analyses produced the same outcome. The de novo assembly performed better in the second round for viroid detection as all the viroids were also detected in 2021 with de novo assembly/BLASTn compared to 2020 when CDVd and HSVd were only detectable via read mapping in samples 2 and 3, respectively. This can be expected as viroid concentration likely increases over time. Read mapping to the non-target CTV genotype T68 and the non-target viroid Cachexia-causing variant of HSVd were in range to be considered as non-target read mapping. As shown in a previous study, non-target read mapping can reach 90% when selecting only a single genotype as a reference [12]. In the present study only two CTV genotypes were selected to concurrently map reads to. The CTV genotype status of the inoculation source was determined previously [7] as CTV genotype RB, and the results obtained correlate with the previous time point. The TPM value rankings per sample based on the Spearman’s rank correlation test of the reference genes also remained the same, but some 1:1 reference gene ratios differed from the initial analysis. This is likely explained by the physiology of the plants as they are in a different developmental stage compared to the first sampling date. However, this had no impact on the accuracy of the virus or viroid detection using HTS but confirms that when reference gene expression values are used for normalization, the stability of reference gene expression should be confirmed for a developmental stage, or a specific tissue type as would be done for any qPCR relative quantitation assay. The HTS pipeline proved to be highly reproducible based on the results obtained in 2021 compared to 2022 despite differences in technicians, reagent lot numbers and plant age between the two time points.

Previous studies have directly compared the ability of HTS and RT-PCR to detect known pathogens in the same sample. In a study comparing detection methods of potato virus A and potato virus Y, small RNA-based HTS was shown to be 10 times more sensitive than real-time RT-PCR [13]. In barley, dsRNA HTS was used and found to have equivalent sensitivity to RT-PCR detection assays [8] and similarly, in fruit trees, dsRNA HTS was found to be equally capable of detecting known viruses and viroids compared to conventional RT-PCR [11]. The few discrepancies observed in these studies were attributed either to the pathogen being below the limit of detection of the RT-PCR, low level of contamination during the HTS pipeline or uneven pathogen distribution [8,11]. In the present study, replicate plants were inoculated from a source plant infected with a known set of pathogens in order for the potential virus infection status to be known. Plants were analyzed at several time points using ribo-depleted RNA HTS and RT-PCR to evaluate the influence of time post-infection, virus/viroid replication rate, virus/viroid rate of accumulation within the host plant, developmental stage of the host plant, and environmental conditions at sampling. The complete virome profile of the source plant was not detected in any of the plants 7 months post inoculation, suggesting a failure in graft transmission of CiVA and CLaf, likely due to uneven distribution of these pathogens or a low concentration in the source plant. Even though the HTS assay applied here successfully detected all pathogens detected with RT-PCR at specific time points, the results highlighted that both techniques are dependent on pathogen concentration for accurate detection and that new infections can potentially be missed if RNA is extracted from newly infected material or a non-representative sample. This was specifically evident with the de novo assembly results of the viroids. Even though one of the major advantages of HTS is the detection of unknown pathogens it is dependent on pathogen concentration in the material selected for the analysis. A combination of de novo assembly and read mapping analyses is recommended for pathogen discovery and routine detection. The RNA extraction method also proved to impact the detection of viroids as more viroid sequences were present in the data when a LiCl free RNA extraction method was used. It is possible that some RNA extraction methods can select against viroid RNA [7]. However, with HTS, more sequencing depth can compensate and increase detection of low concentration pathogens [7,14,15]. The erratic detection of viroid sequences with both RT-PCR and HTS can point to either uneven viroid distribution in the plant, viroid concentration fluctuations during the different time points, or selection against viroid RNA during the RNA extraction method.

The RT-PCR and HTS comparisons performed in this assay was executed independently and therefore could not be influenced by the same cross-over contamination. Only the plant material came from the same greenhouse, but the RNA extractions for the RT-PCR or HTS assays were performed in separate laboratories. This allowed for an independent evaluation of the two pipelines.

## 4. Materials and Methods

### 4.1. Plant Material

To evaluate the reproducibility of the HTS assay, the four plants (*C. sinensis* cv. ‘Madam Vinous’, sweet orange) evaluated in Bester et al. [7], were maintained for an additional year and sampled in January 2021. These four plants were established from seed and included one healthy plant (plant 1) and three plants graft inoculated in February 2019 with a suite of viruses (CTV, CiVA, and CTLV) and viroids (HSVd, CDVd, and CEVd) (plants 2, 3, 4). Three representative leaf samples from each plant were collected (replicate (Rep) sample set).

To evaluate the sensitivity of the HTS assay, two additional sets of plants (six *Citrus sinensis* (L.) Osbeck cv. ‘Madam Vinous’ sweet orange and six *C. aurantiifolia* (Christm.) Swingle cv. ‘Mexican Lime’) were established from seed and five of each cultivar were patch-graft inoculated in May 2020 with a source plant infected with a pre-determined range of pathogens including CTV, CiVA, HSVd, CDVd, CEVd and ‘*Candidatus* Liberibacter africanus’ (CLaf). This source plant (*C. sinensis* cv. ‘Madam Vinous’) was infected in February 2019 as part of the study by Bester et al. [7]. Source and inoculated plants were maintained at CRI in Nelspruit, South Africa in a temperature-controlled greenhouse (24–28 °C) with natural light in summer, but with additional lighting provided in winter months to supply a total of 16 h light per day. At 0, 30, 90 and 203 days post inoculation, two representative leaf samples (RT-PCR sample set and HTS sample set) were taken from the same shoot from each plant to determine the infection status at the various time points.

### 4.2. Total RNA Extraction, RT-PCR and HTS

The Rep sample set was subjected to a Cetyltrimethylammonium bromide (CTAB) extraction protocol (RNA extraction 1) [16].

The RT-PCR sample set of the sensitivity experiment was subjected to RNA extraction and RT-PCR assays as prescribed by the CIS SOP. Briefly, total RNA was extracted from leaves using a protocol utilizing an acid phenol extraction buffer [17] and assayed with RT-PCR as described in Bester et al. [7]. For the HTS sample set, total RNA was extracted using a CTAB extraction protocol [16] (RNA extraction 1). Additionally, a second RNA extract was prepared from the 203 days post inoculation HTS sample set using a different CTAB extraction protocol with acidic phenol phase separation and precipitation with ethanol instead of LiCl (RNA extraction 2) [18,19].

Subsequently a ribo-depleted RNA library was prepared from each sample (72 samples: four ‘Madam Vinous’ samples [7] x three technical replicates, six ‘Madam Vinous’ samples x five extractions, six Mexican Lime samples x five extractions) using the Illumina TruSeq Stranded Total RNA Sample Preparation kit with Plant Ribo-Zero at Macrogen (South Korea). Paired-end HTS (2×100 bp) was performed on an Illumina NovaSeq 6000 instrument (Macrogen, South Korea). The HTS analyses, including quality assessment, de novo assemblies, read mapping, and read count normalization, were performed as described in Bester et al. [7]. Kruskal-Wallis Rank Sum Tests were performed to assess the difference in TPM count ratio between genes. To assess the level of reproducibility between the HTS results obtained in 2021 compared to the results in 2022 a Spearman rank-order correlation test was performed on the TPM values of the reference gene and pathogen sequences. Statistical analyses were performed in R 4.0.2 available from the Comprehensive R Archive Network (CRAN).

## 5. Conclusions

The sensitivity experiment in this study proved that HTS can be a reliable and comparable assay to RT-PCR. However, an HTS assay will have its own limit of detection influenced by pathogen concentration, sample processing method and sequencing depth. In most of the previous studies plants were evaluated at one time point and without background knowledge of the expected virome profile. In this study the expected virome profile was known and plants were evaluated over time to allow for pathogen accumulation in the plant. The success of graft transmission from the source plant was a variable that could not be controlled and the detection of any of the expected pathogens in the inoculated plants was scored as positive irrespective of whether it was detected in the RT-PCR or HTS data set. However, in a real scenario, it will be unknown which virus/viroid to expect and therefore to decide which method between RT-PCR and HTS is correct or more sensitive if conflicting results are obtained. It is therefore essential to validate an approach to minimize the risk of false-negative and false-positive results. The limit of detection of PCR and HTS assays will differ and factors such as sample processing, method and reagents used will impact differently on an RT-PCR and HTS assay. The detection of different pathogens will also be differentially affected by these factors and therefore each detection method needs to be evaluated for each specific host/pathogen/assay combination to be endorsed as a repeatable, specific, sensitive, and reproducible detection assay. In this study, we directly compared an HTS assay to standard RT-PCR assays for the accurate detection of CTV and three viroids in citrus and found that the HTS assay is equivalent.

## Data Availability

The data presented in this study are available in Supplementary Files S1, S2, and S3.

## Supplementary Materials

The following supporting information can be downloaded at: https://www.mdpi.com/article/10.3390/plants11151939/s1, Supplementary File S1: Descriptive statistics of high-throughput sequencing data generated and the identification of contigs/scaffolds per de novo assembly for data obtained in 2020 and 2021.; Supplementary File S2: Spearman’s rank correlation test performed on the transcripts per million (TPM) values obtained for each reference gene and pathogen sequence analyzed in data obtained in 2020 and 2021 from the same plants to assess the level of reproducibility of an HTS assay.; Supplementary File S3: Descriptive statistics of high-throughput sequencing data generated and the identification of contigs/scaffolds per de novo assembly for sensitivity experiment.
